# Biomarkers of Coagulation and Fibrinolysis during Cemented Total Hip Arthroplasty with Pre- versus Postoperative Start of Thromboprophylaxis

**DOI:** 10.1155/2013/563217

**Published:** 2013-12-16

**Authors:** Pål O. Borgen, Ola E. Dahl, Olav Reikeras

**Affiliations:** ^1^Department of Orthopaedics, Martina Hansens Hospital, Postboks 23, 1306 Baerum Postterminal, Norway; ^2^Innlandet Hospital Trust, 2381 Brumunddal, Norway; ^3^Thrombosis Research Institute, London SW36LR, UK; ^4^Department of Orthopaedics, Oslo University Hospital, Rikshospitalet, N-0027 Oslo, Norway

## Abstract

Venous thrombosis is common in elective hip surgery, and prophylaxis is recommended. Clinical trials suggest that the drug dose and timing of initiating prophylaxis significantly influence antithrombotic effectiveness and safety. We studied the time course and gradient of plasma coagulation and fibrinolysis during total hip arthroplasty (THA) in twenty patients that were randomly assigned to have the first dose of 5000 IU dalteparin subcutaneously (sc) injected 12 hours before or 6 hours after surgery. Baseline characteristics were similar in both groups. Specific biomarkers on coagulation (prothrombin fragment 1+2 (F1+2)) and fibrinolytic activity (plasmin/**α**2-antiplasmin complex (PAP) and D-dimer) were collected at six events during hospitalization and analysed. There were no significant group differences in the biomarkers at any time point. The highest concentrations were measured 6 hours after surgery and before the first postoperative injection. A marked decrease followed at the first postoperative day, and then a second increase in plasma concentrations was observed 6 days after surgery. This study showed that activation of coagulation and fibrinolysis by the operative trauma was the same when the first dose of dalteparin was injected 12 hours before or 6 hours after surgery.

## 1. Introduction

Thrombosis formation begins during joint replacement surgery [1, 2], and a few patients may develop nonfatal or fatal pulmonary embolism (PE) [3]. It has been suggested that it is easier to prevent thrombus formation than to arrest thrombus growth once it has been established. Preoperative initiation of thromboprophylaxis therefore has been recommended [4, 5]. However, most thrombi develop postoperatively [6, 7], and, because anticoagulants have the potential to increase bleeding, some surgeons and anesthesiologists prefer postoperative initiation to reduce blood loss, need for transfusion, and bleeding complications [8–10]. Low-molecular-weight heparins (LMWHs) are widely used as antithrombotic because of their favorable efficacy-to-safety profiles and the absence of accumulated postmarketing reports on severe adverse events. Trials on timing of thromboprophylaxis have been designed to detect thrombotic events, and venographically detected DVT has been the primary end point. Bleeding has been a secondary underpowered outcome, and trials have been criticized for underestimating the risk of bleeding and related complications [11]. From surgeons point of view, blood loss and bleeding complications are important and pharmaceutical prophylaxis has remained controversial [12, 13].

There are no head to head comparisons with different regimens using the same drug; therefore, both preoperative and postoperative initiations of prophylaxis are still recommended in recent guidelines [14], and the need for further investigations has been emphasized. In a retrospective study on patients undergoing total hip arthroplasty (THA), we found reduced bleeding when dalteparin prophylaxis was started after surgery [15]. This was not confirmed in a prospective, randomized double blind clinical study where an identical dose of dalteparin administered 12 hours before or 6 hours after THA caused the same volume of blood loss and bleeding related events in both groups [16]. This finding was newly substantiated in another study on knee replacement patients [17]. The biochemical rationale for this finding is uncertain and needs to be clarified.

Several biomarkers have been used to study haemostatic response to surgery [18, 19]. Furthermore, they have been proposed as surrogate endpoints of bleeding and venous thromboembolism (VTE) and to be of prognostic value to assess clinical outcome [20, 21]. F1+2 fragment is produced when prothrombin is converted to thrombin which acts on fibrin to form blood clots [22], while plasmin/*α*2-antiplasmin complex (PAP) and D-dimer have been found to be valuable markers of fibrinolytic activity during THA [23].

In this present study, we measured changes in these haemostatic markers to assess potential alterations when thromboprophylaxis was initiated with 5000 IU dalteparin injected 12 hours before versus 6 hours after THA surgery. Based on our clinical randomized study with no recorded differences in blood loss, bleeding events, and thromboembolic events, we hypothesized that activation of these haemostatic markers is the same in pre- versus postoperative start of prophylaxis. The results of these plasma analyses are presented here.

## 2. Material and Methods

The material consisted of THA patients included in a clinical prospective randomized double blind study on safety and efficacy of preoperative versus postoperative initiated thromboprophylaxis conducted at Martina Hansens Hospital between March and June 2008. The study was approved by the Regional Ethics Committee (08012d), registered in the Norwegian Biobank register (2058), and performed in accordance with the ethical standards of the Declaration of Helsinki.

International Normalization Ratio (INR) without thromboprophylaxis is normally approximately 1.0. In patients on anticoagulants, a level of 1.8 (which is 80% higher) is generally accepted for performing spinal anesthesia and major orthopedic surgery. Without previous data on the effect of dalteparin versus placebo on these biomarkers, we calculated the sample size using previously published data on F1+2 during THA surgery [24]. To detect an 80% difference in the increase in F1+2 with or without dalteparin, 10 patients in each group would have the power of 80% with an alpha of 0.05.

After signing informed consent, 20 patients above 50 years that underwent primary cemented THA due to osteoarthritis were randomly allocated to either 12 hours preoperative or 6 hours postoperative start with 5000 IU dalteparin (Fragmin, Pharmacia and Upjohn, Stockholm, Sweden). All patients received spinal anesthesia without hypotensive effect with 5 mg/mL bupivacaine (Marcain; AstraZeneca, Södertälje, Sweden) injected at the lumbar level. Cephalothin (Keflin; EuroCept Pharmaceuticals BV, Kortenhoef, The Netherlands) at 2 g × 4 was given intravenously as prophylaxis against infection. Voluven and Ringer's acetate (Fresenius KABI, Bad Homburg, Germany) were used as plasma substitutes.

The operation was performed in the lateral position, using a standardized posterior approach where only the piriform muscle was detached and with capsular repair at the end of the procedure. Postoperative analgesia was administered according to a standard protocol consisting of paracetamol + codeine sulfate (Paralgin forte; Weifa AS, Oslo, Norway) and ketobemidone (Ketorax; Jenahexal Pharma, Jena, Germany). Closed postoperative drainage was used for 24 hours. All patients were mobilized on the first postoperative day. We did not allow concomitant mechanical prophylaxis against DVT.

Patients with allergy to LMWH, bleeding disorders, renal failure, hepatic disease, active treatment for malignancy, ongoing antithrombotic treatment, and history of DVT or PE and patients experiencing major operations, traumas, stroke, or cardiac infarction the last 3 months before surgery were excluded. In the hospital's written patient information, patients were advised to stop antiplatelet medication, that is, NSAIDs and high-dose aspirin, 1 week before surgery.

We assigned patients to either 5000 IU dalteparin subcutaneously or placebo (saline) injected 12 hours before surgery. All patients had 5000 IU dalteparin subcutaneously 6 hours after surgery and each day until the 35th postoperative day. The syringes with 5000 IU dalteparin and placebo with the same volume in each syringe were prepared by a study nurse who otherwise was not engaged in the study, according to randomized strata. The injection was blinded to the investigator, hospital staff, and the patient. The study blinding was broken after all patients had completed 6 months' follow-up. No patients were lost to follow up.

Haemoglobin (Hgb.), haematocrit (Hct.), white blood counts (WBC), platelet counts (PLT), C-reactive protein (CRP), creatinine (Cr), and liver enzymes were analysed the day before surgery.

Blood samples for biomarkers were obtained from peripheral veins at the following time points: (T1) day before surgery, (T2) before induction of anaesthesia, (T3) at the end of surgery, (T4) 6 hours after surgery and before injection of dalteparin, (T5) the day after surgery, and (T6) 6 days after surgery. Blood sample was kept on ice until it was separated by centrifugation at 2500 g for 20 min at 18 degrees C and stored at −80 degrees C until assayed.

### 2.1. Laboratory Analyses

Prothrombin fragment F1+2 was measured in citrated plasma by ELISA using a commercial kit (Enzygnost F1+2 micro, Dade Behring, Marburg, Germany), following manufacturer's instructions. Plasmin/*α*2-antiplasmin (PAP) complex was measured in citrated plasma by ELISA using a commercial kit (Enzygnost PAP micro, Dade Behring, Marburg, Germany) following manufacturer's instructions. D-dimer was determined in citrated plasma using a commercial kit (STA-Liatest D-Di, Diagnostica Stago, Asnières s/Seine, France) following the manufacturer's instructions.

### 2.2. Statistical Analyses

Statistical analyses were performed using SPSS II software Version 19 (IBM Inc., USA). Data are presented by mean and standard deviation. Independent samples *t*-test is used to compare descriptive variables. Time dependent changes were performed by two-way analysis of variance (ANOVA). If significant differences were indicated, we used the LSD post hoc test. *P* ≤ 0.05 was considered significant.

## 3. Results

Baseline patient characteristics were similar in the two groups (Table 1).

There were no significant group differences of F1+2, PAP, and D-dimer at any time point (Tables 2, 3, and 4). No significant changes in biomarkers were demonstrated from 12 hours before until start of surgery. Surgery caused marked increases in F1+2, PAP, and D-dimer synchronously in both groups. The highest concentrations were measured 6 hours after surgery before the first postoperative dalteparin injection where after they declined to lowest level on day 1. Between postoperative day 1 and 6, modest increases in F1+2 and D-dimer were recorded, while the level of PAP was significantly increased (*P* = 0.006 and 0.001 in preoperative and postoperative group).

Clinically, one patient in the preoperative group experienced hematoma which was evacuated during hospitalization. There were no thromboembolic events.

## 4. Discussion

In this study based on a prospective randomized double blind study with pre- versus postoperative initiation of the same dose of dalteparin, markers on coagulation and fibrinolysis showed that intravascular thrombin formation (F1+2,) and plasmin activity (PAP and D-dimer) increased almost simultaneously during surgery, reached maximum 6 hours postoperatively, and declined the next 12 hours. All the biomarkers were significantly higher at the end of the first postoperative week than those before surgery (Tables 2, 3, and 4). Preoperative or postoperative dalteparin administration did not change this hemostatic pattern. This variation in pro- and anticoagulant activities over time is in accordance with other studies [2]. It also confirmed the primary endpoint, that is, the bleeding parameters in our clinical trial that showed the same bleeding whether 5000 IU dalteparin was injected 12 hours before or 6 hours after surgery [16]. The results are also in accordance with a recent study by Llau [17] and colleges who injected 40 mg enoxaparin at the same timepoints after total knee arthroplasties (TKA).

There are some limitations to this study. At all time points, there were marginal differences in F1+2 between the two groups, and we are aware that with a small number of patients these differences might have been significant if the patient number was increased. However, to reach statistical significant differences between these treatment groups, the number of patients had to be over 400 in each group, which indicate that this difference is of no clinical significance, and, from an ethical point of view, an expansion of the study population would have been questionable.

We collected blood from peripheral veins. Earlier studies have demonstrated a more moderate expression of the level of biomarkers in peripheral venous blood compared to arterial blood or mixed venous blood, which may be due to passage of the arteriovenous filter or dilution [25]. Furthermore, several biomarkers are available to analyze coagulation and fibrinolysis and they reflect activity from different parts of these processes. The selected biomarkers might not be the optimal ones to measure the influence of dalteparin on hemostasis during surgery.

The various LMWHs differ in their pharmacokinetic properties and anticoagulant activity [26], and, even if others have shown the same clinical pattern [16], the results of this study should not be generalized for other compounds.

The levels of biomarkers were similar at baseline and before surgery although only one group had preoperative dalteparin. This could be expected, as hemostasis was not yet activated. However, lack of group differences during and after surgery was not anticipated since preoperative administered dalteparin was thought to neutralize thrombin activity [4, 19]. An explanation might be that the substantial thrombin generation (F1+2) caused by the operation masked the remaining effect of dalteparin injected 12 hours before surgery due to its bioavailability with a half-life of 3-4 h [26, 27].

The sharp increase in all biomarkers recorded during surgery reflects that THA surgery, which involves mechanical obstruction of veins in the lower extremities, endothelial damage, and destruction of bone marrow, is a strong signal for hemostatic activity. These observations harmonize with others [1]. The level of quantified biomarkers continued to increase after surgery and peaked at 6 hours which probably is the result of haemostatic amplification when the blood passes the lung circulation [2]. After the first postoperative dalteparin injection 6 hour postoperatively and until the day after surgery, we recorded a rapid decrease in this activity in both groups. These observations concur with previous findings that fibrinolytic activity is enhanced intraoperatively with a shutdown after surgery [2, 28].

During major surgery, there is a complex interaction of cellular components and pro- and anticoagulant factors, to form stable clots. The dynamic of blood loss, dilution, and consumption of these haemostatic factors may lead to the observed reduction of biomarkers on day 1 after surgery. Natural variations during the day and increased plasminogen activator inhibitor (PAI) activity have also been proposed as explanations for this “fibrinolytic shut-down.” Alternatively it may simply be dalteparin inhibition of Factor Xa and thrombin. Plasma PAP reflects clot formation and fibrin degradation and is regarded as an index of recent fibrinolytic activity [29]. Results from previous investigations with other biomarkers indicated that decreased fibrinolytic activity was associated with thromboembolism after surgery [20, 21]. The data from these studies are consistent with the PAP pattern in our study. These authors have also showed that the referred biomarker plasma levels were unaffected by anticoagulation during THA surgery, which is in line with our findings. F1+2 activity in the present study paralleled the fibrinolytic activity and was also unaffected of LMWH.

The observed profile of high or increasing levels of these biomarkers both from baseline and from the first postoperative day until the 6th postoperative day in our series harmonizes with others and indicates a continuing procoagulant state even beyond hospital discharge in several patients [18, 30].

Increased plasma concentrations of F1+2 and D-dimer are found to correlate with thrombosis, but with relative low specificity and predictability [31, 32]. Previously, we have reported the same amount of bleeding with the two regimens [16]. LMWHs have repeatedly been shown to be effective against postoperative thrombosis after THA, and our findings therefore support the view that dose and the interval between surgery and the first administration of prophylaxis are important [9, 33].

The results from clinical investigations on timing of prophylaxis have been divergent. Bergqvist [4] and colleges showed reduced incidence of DVT and increased bleeding when 5000 IU dalteparin compared to half the dose was injected before surgery and pointed out that effect of dalteparin was dose dependent even if it was administered the day before surgery. The majority of his patients had abdominal procedures known to stimulate less thrombotic activity than THA, which can explain why our laboratory results do not concur with his observations.

Hull et al. [33] observed increased protocol defined major bleeding when dalteparin was injected within 2 hours preoperatively, compared to administration 12 hour preoperatively or warfarin 24 hours postoperatively. However, the recorded perioperative volume of blood loss did not differ markedly. In our prospective controlled clinical trial, we could not demonstrate difference in blood loss or bleeding events when dalteparin was initiated 12 hour before or 6 hours after surgery. The present observation with no difference in hemostatic biomarkers is in harmony with our clinical observations.

## 5. Conclusion

Our hemoanalyses confirms that activation of thrombin generation and fibrinolysis starts during THA surgery. No difference in activation pattern was demonstrated comparing pre- versus postoperative initiation of thromboprophylaxis with dalteparin.

## Figures and Tables

**Table 1:** Patient characteristics (mean ± standard deviation) and *P* value.

Characteristic	Preoperative group	Postoperative group	*P* value
Number of patients	10	10	
Sex (% males)	30	50	
Age (years)	65.6 ± 6.9	71.2 ± 6.6	0.083
Height (cm)	168.0 ± 8.7	171.5 ± 9.4	0.397
Weight (kg)	73.8 ± 16.8	81.9 ± 15.8	0.282
BMI (kg/m²)	26.1 ± 5.3	28.0 ± 0.6	0.453
ASA classification	1.9 ± 0.6	2.0 ± 0.7	0.722
Preop. hemoglobin	14.3 ± 0.9	14.0 ± 0.7	0.495
Preop. hematocrit	41.9 ± 3.5	40.7 ± 2.5	0.395
Preop. C-reactive protein	2.6 ± 3.0	3.9 ± 5.6	0.540
Preop. creatinine	58.6 ± 10.6	66.0 ± 11.0	0.142
Preop. blood plates	246 ± 93.1	246 ± 56.6	1.0

**Table 2:** F1+2 (pmol·mL^−1^). Time points are the day before surgery (1), after anaesthesia but before surgery (2), at the end of wound closure (3), at 6 hours after surgery (4), at the first day after surgery (5), and at 6 days after surgery (6). Values are mean ± standard deviation (SD) and 95% confidence interval (CI).

Time point	Preop. group	Postop. group	*P* value (ANOVA)
T1	214 ± 63 (131–297)	212 ± 99 (129–295)	0.799
T2	184 ± 56 (101–267)	148 ± 67 (65–231)	0.799
T3	532 ± 148^a^ (449–615)	567 ± 187^e^ (484–649)	0.799
T4	594 ± 173^b^ (512–677)	549 ± 131^f^ (466–632)	0.799
T5	250 ± 140^c^ (167–333)	310 ± 194^g^ (227–393)	0.799
T6	335 ± 115^d^ (252–418)	362 ± 113^h^ (279–445)	0.799

*P* value (ANOVA)	<0.001	<0.001	

^a^ *P* < 0.001; ^b^ *P* < 0.001; ^c^ *P* = 0.240; ^d^ *P* = 0.009; ^e^ *P* < 0.001; ^f^ *P* < 0.001; ^g^ *P* = 0.012; ^h^ *P* = 0.001, all in relation to time point 2 (before surgery).

**Table 3:** D-dimer (*μ*g·mL^−1^). Time points are the day before surgery (1), after anaesthesia but before surgery (2), at the end of wound closure (3), at 6 hours after surgery (4), at the first day after surgery (5), and at 6 days after surgery (6). Values are mean ± standard deviation (SD) and 95% confidence interval (CI).

Time point	Preop. group	Postop. group	*P* value (ANOVA)
1	0.76 ± 0.47 (0.02–1.51)	0.69 ± 0.49 (−0.6–1.4)	0.965
2	0.75 ± 0.56 (0.001–1.49)	0.79 ± 0.87 (0.04–1.53)	0.965
3	3.71 ± 1.22^a^ (2.97–4.46)	4.24 ± 1.72^e^ (3.49–4.98)	0.965
4	5.15 ± 2.19^b^ (4.40–5.89)	4.80 ± 1.78^f^ (4.05–5.54)	0.965
5	2.61 ± 1.15^c^ (1.87–3.35)	2.41 ± 0.89^g^ (1.66–3.15)	0.965
6	1.97 ± 0.42^d^ (1.19–2.76)	2.09 ± 0.69^h^ (1.3–2.87)	0.965

*P* value (ANOVA)	<0.001	<0.001	

^a^ *P* < 0.001; ^b^ *P* < 0.001; ^c^ *P* = 0.001; ^d^ *P* = 0.029; ^e^ *P* < 0.001; ^f^ *P* < 0.001; ^g^ *P* = 0.004; ^h^ *P* = 0.021, all in relation to time point 2.

**Table 4:** PAP (*μ*g·L^−1^). Time points are the day before surgery (1), after anaesthesia but before surgery (2), at the end of wound closure (3), at 6 hours after surgery (4), at the first day after surgery (5), and at 6 days after surgery (6). Values are mean ± standard deviation (SD) and 95% confidence interval (CI).

Time point	Preop. group	Postop. group	*P* value (ANOVA)
1	627 ± 153 (510–744)	511 ± 172 (394–628)	0.110
2	616 ± 149 (499–733)	478 ± 106 (361–595)	0.110
3	917 ± 257^a^ (800–1034)	936 ± 255^e^ (819–1053)	0.110
4	1084 ± 326^b^ (967–12019)	1033 ± 204^f^ (916–1151)	0.110
5	588 ± 124^c^ (471–705)	539 ± 97^g^ (422–656)	0.110
6	846 ± 90^d^ (729–963)	851 ± 135^h^ (734–968)	0.110

*P* value time (ANOVA)	<0.001	<0.001	

^a^ *P* = 0.002; ^b^ *P* < 0.001; ^c^ *P* = 0.756; ^d^ *P* = 0.013; ^e^ *P* < 0.001; ^f^ *P* < 0.001; ^g^ *P* = 0.429; ^h^ *P* < 0.001, all in relation to time point 2.
